# Coordination Polymer Framework-Derived Ni-N-Doped Carbon Nanotubes for Electro-Oxidation of Urea

**DOI:** 10.3390/ma15062048

**Published:** 2022-03-10

**Authors:** Vadahanambi Sridhar, Hyun Park

**Affiliations:** 1 Global Core Research Centre for Ships and Offshore Plants (GCRC-SOP), Pusan National University, Busan 46241, Korea; sridhar@pusan.ac.kr; 2 Department of Naval Architecture and Ocean Engineering, Pusan National University, Busan 46241, Korea

**Keywords:** coordination polymer frameworks, water splitting, oxygen reduction reaction, urea, microwave synthesis

## Abstract

Electrochemical oxidation of urea (UOR) is critical in the removal of urea from wastewater and energy conservation and storage. Nickel-based catalysts are widely used for urea-ORR, but in all cases, the nickel must be hybridized with carbon materials to improve its conductivity. In this manuscript, we demonstrate the synthesis of a nickel-decorated carbon nanotube (Ni-NCNT) by simple microwave pyrolysis of Dabco (1,4-diazabicyclo[2.2.2]octane)-based coordination polymer frameworks (CPF). The surface structure, morphology and chemical composition of Ni-NCNT were characterized by Raman spectrum, scanning electron microscopy (SEM), transmission electron microscopy (TEM), X-ray photoelectron spectroscopy and energy-dispersive X-ray spectroscopy (EDS) analysis. SEM studies showed micrometer-long bamboo-shaped CNTs with nickel nanoparticles anchored to the walls and inside the nanotubes. A structural study by TEM and Raman spectra showed that carbon nanotubes are rich in defects due to the presence of nitrogen, and this was confirmed by energy-dispersive X-ray spectroscopy (EDS) maps. When applied as electrocatalysts in urea oxidation reactions (UOR), our newly developed Ni-NCNT shows excellent electrocatalytic activity and stability, making it a versatile catalyst in energy generation and mitigating water contamination.

## 1. Introduction

With increasing urbanization, there is a tremendous increase in the release of urea-rich wastewater, which is harmful to the environment and public health. Besides urban households, urea-rich wastewaters are also generated from urea-based industries (urea-formaldehyde resins and explosives) and agricultural industries [1,2]. The ever-increasing usage of urea and phosphate-based fertilizers causes “eutrophication” (enrichment of surface waters with plant nutrients), which results in excessive plant and algal growth [3]. Besides water pollution, hydrolysis of urea causes ammonia volatilization, which leads to breathing problems [4]. On the other hand, urea is receiving considerable attention in the fuel cell sector since urea is a promising hydrogen carrier that can be directly fed into direct urea fuel cells (DUFC). Urea is stable and widely available when compared to other hydrogen carriers and liquid fuels like water, methanol, formic acid, ethanol and glycerol [5,6]. Moreover, urea electrolysis is considered more effective and energy-efficient when compared to water electrolysis [7,8,9]. When compared to other liquid fuels, urea possesses excellent oxidation thermodynamics, and the primary urea oxidation reaction (UOR) and the half-wave potential determine the performance of DUFC. The electrochemical technique is very promising for urea oxidation and elimination; however, the reaction kinetics of urea oxidation are relatively slow due to the so-called ‘six-electron transfer process’ occurring at the anode [10,11]. Noble metal catalysts such as Pt [12,13] and Ru [14]-based catalysts can improve the catalytic performance of urea oxidation, but their high cost and scarcity limit their large-scale industrial application, which necessitates the need to find more efficient and cost-effective electrocatalysts for UOR. 

Amongst the various non-noble metal alternatives like copper [15], cobalt [16], iron [17], etc., it was found that nickel-based non-noble metal catalysts showed better electrocatalytic activity for urea electro-oxidation [18,19,20]. Pure nickel-based electrocatalysts in UOR reactions have serious drawbacks like sluggish reaction kinetics, requirement of large overall potential and poor stability [21,22]. Recently, some researchers have improved the activity and stability of nickel-based catalysts by either morphological structure tuning using non-metal elements like boron, phosphorous, nitrogen, sulfur [23] to dope nickel and change its electronic properties or by alloying nickel with metals to form nickel alloys like NiFe [24], NiW [25], NiMo [26], NiCu [27], etc. However, there is one thing common in all of the abovementioned techniques, which is the necessity of hybridizing nickel-based electrocatalysts with conductive carbon materials like carbon fiber [28], carbon nanotubes [29], graphene [30], etc. In most cases, this is done by ex situ methods wherein pre-synthesized nickel-based electrocatalysts are hybridized with pre-treated carbon materials to form relatively stable covalently bonded nickel-carbon hybrids. However, the ex situ method involves multiple steps and, more importantly, as the name indicates, relies on the substitution of nitrogen moieties in the defects on carbon surfaces. Additionally, it is generally known that defects in graphene are more prevalent on the edges when compared to the bulk. Therefore, in order to overcome these drawbacks, we developed a simple technique to synthesize nickel-nitrogen-doped carbon nanotube hybrids by fast and facile microwave pyrolysis of Dabco (1,4-diazabicyclo[2.2.2]octane)-based nickel coordination polymer frameworks (CPF). Although CNTs are traditionally synthesized by chemical vapor deposition CVD using separate sources for catalysts and carbons (usually hydrocarbon gases or vaporized hydrocarbon liquids), our aim is to develop single sources containing both the catalyst and the carbon source for the synthesis of CNT. This manuscript is part of our series of papers on the investigation of single-source carbon nanotube precursors, wherein we have previously reported the synthesis of CNTs from zeolitic imidazolate frameworks (ZIF) [31] and benzene-1,3,5-carboxylic acid [32]-based metal organic framework (MOF) precursors. 

## 2. Experimental

### 2.1. Materials and Methods 

Reagent-grade chemicals nickel acetate (CAS Number: 373-02-4, ACS reagent grade, 98% purity), Dabco (CAS Number: 280-57-9, ACS reagent grade, 99% purity) were purchased from Sigma-Aldrich, Seoul, Korea, whereas urea (CAS Number: 57-13-6, ACS reagent grade, 98% purity) was purchased from Alfa Aesar, Seoul, Korea and were used as received. Microwave synthesis was carried out in a microwave oven, Model number: KR-B202WL, manufactured by Daewoo, Seoul, Korea, operated at 700 W and 2450 MHz frequency. Morphology of the Ni-NCNT was studied by a field-emission scanning electron microscope (SEM) manufactured by Zeiss, Seoul, Korea, FEG-SEM Supra 25, operated at 10 kV and no coating was applied in SEM tests due to the inherent high electric conductivity of our Ni-NCNT samples. High-resolution transmission electron microscopic (HRTEM) images, high-angle annular dark-field (HAADF) images and elemental maps were recorded on a TALOS F200X, manufactured by Thermo Fisher Scientific Korea Ltd., Seoul, Korea and operating at 200 kV. Ni-NCNT was drop-casted onto TEM grids after ultrasonicating them in ethanol solution for 120 s. Chemical moieties were studied by a Sigma Probe Thermo VG X-ray photoelectron spectrometer (XPS). Deconvolution and curve fitting of XPS data was carried out using XPSPEAK 4.1. Thermogravimetric analysis (TGA) was carried out on PerkinElmer, STA 6000 manufactured by PerkinElmer, Waltham, MA, USA in a nitrogen atmosphere at a heating rate of 5 °C min^−1^.

### 2.2. Synthesis of Ni-NCNT from CPF Precursors

The synthesis of Ni-NCNT from CPF precursors involved two steps; the first step was the synthesis of Dabco-based Ni-CPF and its subsequent transformation to Ni-NCNT under microwave radiation. Nickel acetate (11.24 mmol) and 5.52 mmol of DABCO were added to methanol, and the mixture was ultrasonicated for 30 min and dried in a vacuum oven at 90 °C to remove methanol to yield solid Ni-CPF. Representative in-lens and secondary electron SEM micrographs of Dabco-based Ni-CPF are exhibited in Figure S1a,b of the supplementary file. This powder was subsequently transferred into a graphite crucible and subjected to microwave radiation for 45 s at 700 W to yield a fluffy powdery solid nickel-nitrogen-doped carbon nanotube hybrid.

### 2.3. Electrochemical Measurements 

Electrochemical testing was carried out in a conventional three-electrode system using a Bio-Logic electrochemical workstation, manufactured by Biologic, Seyssinet-Pariset, France. A working electrode was prepared by coating a catalyst ink on a glassy carbon electrode (3.0 mm in diameter) using 5% w/w in water and 1-propanol as the binder to stabilize the catalyst. Before the start of every electrochemical test, the glassy carbon electrode was thoroughly cleaned and polished with 1 μm and 100 nm alumina powder and then finally sonicated in ultrapure water for a few seconds and dried naturally. A reversible hydrogen electrode (RHE) was used as the reference electrode, and a graphite rod was used as the counter electrode. The catalyst ink for fabrication of the working electrode involved mixing 5 mg catalyst, 50 μL Nafion solution and 450 μL ethanol in a sonicator for 30 min to form a uniform catalyst suspension. Then, 20 μL of the catalyst suspension was added drop-wise to the glassy carbon electrode, dried naturally and was used for electrochemical testing as a working electrode. Cyclic voltammetry (CV) was used to evaluate the catalytic performance at various scanning rates of 1 mV s^−1^ to 10 mV s^−1^ for urea oxidation, and the electrolyte was 1 M KOH or 1 M KOH/0.33 M urea. 

## 3. Results and Discussion

**Figure 1:**
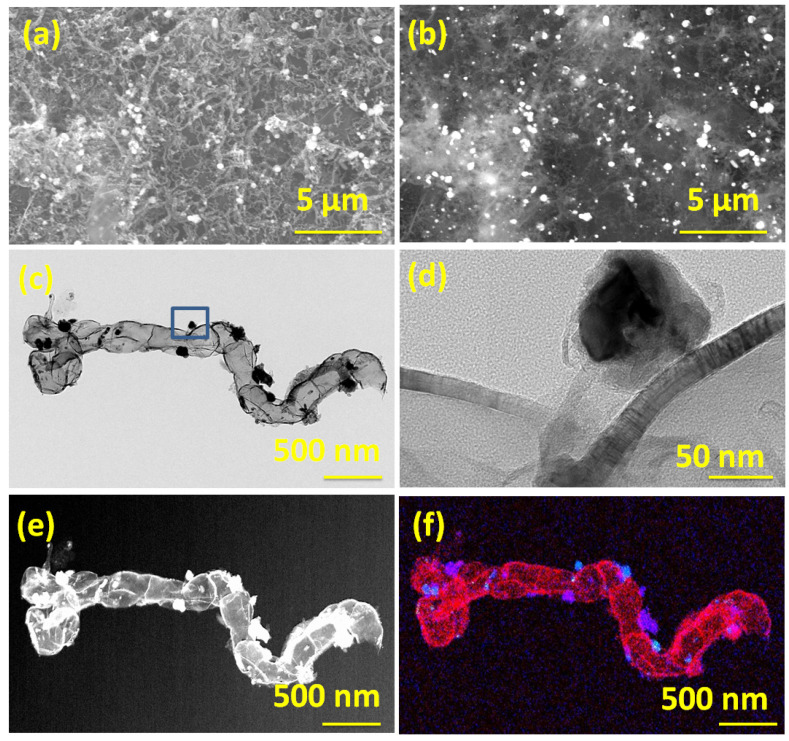
In-lens (**a**) and secondary electron emission (**b**) SEM micrographs of Ni-NCNT; Transmission electron micrographs TEM (**c**) and HRTEM (**d**) of Ni-NCNT and its corresponding dark field (**e**), elemental composite map (**f**). Scale bars are 5 µm in (**a**,**b**); 500 nm in (**c**,**e**,**f**) and 50 nm in (**d**), respectively. Blue color in (**f**) shows nickel moieties, and the red color shows nitrogen moieties.

The morphology and microstructure of Dabco Ni-CPF-derived carbon nanotubes as studied by in-lens SEM (Figure 1a) showed micrometer-long irregularly-shaped non-straight carbon nanotubes. The corresponding secondary electron SEM images exhibited in Figure 1b show nano-dimensional nickel particles anchored on the walls of the carbon nanotubes. The representative TEM micrographs in Figure 1c confirm the irregular-shaped micrometer-long carbon nanotubes. Additionally, most of the nickel nanoparticle catalysts were either enclosed in carbon nanotubes or anchored on the surface of the graphene substrate. Careful high-resolution TEM analysis of CNTs (Figure 1d) revealed that the nanotubes had partitioned, but inter-connected hollow compartments and the walls of the tubes were curved with defects in the outermost few layers, whereas the inner walls were defect-free and irregular shaped nickel nanoparticles were core-shell structured with thin carbon coating. The shells were uniform in thickness and estimated to be in the range of 27–32 layers, with the outer 3–4 layers showing a d-spacing of 0.36 nm (indicating partial exfoliation), whereas the remaining inner layers had a d-spacing of 0.34 nm, typical of graphite (002) planes. The nickel nanoparticles were crystalline and had lattice fringe spacing of ~0.204 nm, which is close to that (111) of pristine nickel [33]. The mechanism of transformation of Dabco-based Ni-CPF to Ni-NCNT can be explained in two concurrent steps. During microwave radiation, eddy currents are generated when a conductive material (Dabco-based Ni-CPF in this case) is exposed to an inter-changing magnetic field due to the relative motion of the field source and conductor or due to variations of the field with time [34]. These eddy-currents lead to Joule heating of the material, and especially in the case of nano-sized magnetic materials, like Ni-CPF, the heating will be intense and instantaneous. This joule heating results in the released nitrogenated hydrocarbons of H_2_NCH_2_CH_2_ [35], which are captured by nickel moieties and result in the formation of nitrogen-doped carbon nanotubes via diffusion of carbon through the Ni particle [36]. This is reflected in the elemental map shown in Figure 1f (and its corresponding dark field image (Figure 1e)), wherein the nickel moieties in blue are predominantly present on the walls of the carbon nanotubes whereas the nitrogen moieties are on the walls of the ‘compartments’. 

**Figure 2:**
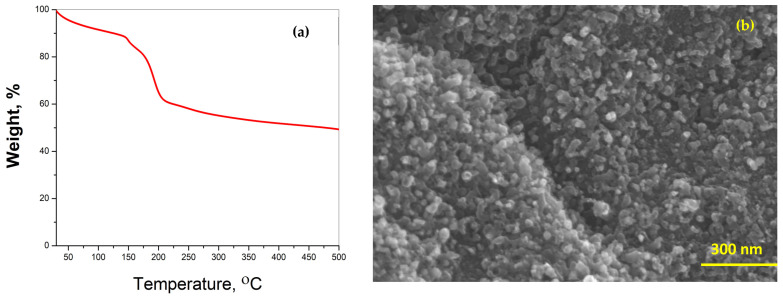
TGA thermograph (**a**) and SEM morphology (**b**) of ‘ash residue’ after TGA experiment.

Thermogravimetric analysis (TGA) was performed at a heating rate of 5 °C min^−1^ to determine the thermal stability of Dabco-synthesized Ni-CPF in a nitrogen atmosphere (Figure 2a). The TG curves showed three distinct regions: the first mass loss of 12% between 80 °C and 120 °C attributed to the evaporation of water molecules; the area between 125 °C and 220 °C, exhibiting a rapid mass loss of 29.6% related to the decomposition of organic moieties of Dabco and its reaction with nickel; and the third slow but steady mass loss starting at 220 °C can be attributed to the total collapse of CPF and its disintegration to nickel-rich carbons. There have been many reports on TGA of reticular porous nanostructures, but the morphological study by SEM of the ‘ash residue’ after TGA experiments is seldom investigated, at least in the case of Dabco-based CPFs, it has not been reported to date. This manuscript is the first-ever report on the SEM morphology of ‘residual ash’ of Dabco-based CPF exhibited in Figure 2b. Though SEM morphology shows the formation of carbon-rich tubular structures, the tubes are substantially smaller when compared to the microwave-obtained NCNTs. This proves that microwave energy is essential for the proper formation of carbon nanotubes from Dabco-based CPF precursors.

**Figure 3:**
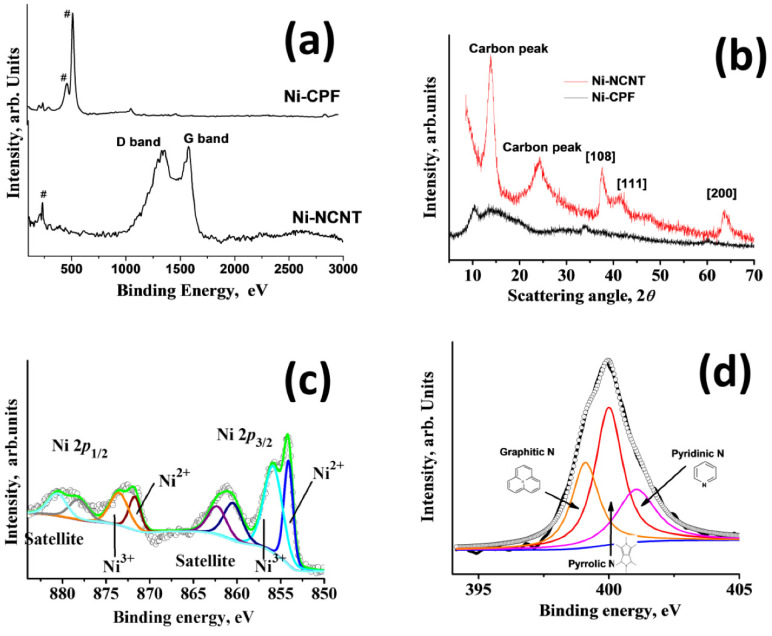
Raman spectra (**a**) and XRD patterns (**b**) of Ni-CPF and Ni-NCNT; deconvoluted Ni 2p (**c**) and N 1s (**d**) XPS scans of Ni-NCNT.

Raman spectra were carried out to study the structural properties of Ni-CPF and Ni-NCNT (Figure 3a), wherein the peaks associated with nickel are marked with #. In the case of Ni-NCNT, three describable peaks can be observed: one associated with nickel at 208 cm^−1^, attributed to the T2g symmetry; and two associated with the Raman active G and D band peaks at 1584 and 1352 cm^−1^, attributed to the in-plane vibrational mode and disorder induced by the presence of extensive defects, as observed in HRTEM in Figure 1d. In the case of Ni-CPF, the carbon-related Raman active peaks were absent, and the spectrum was dominated by intense peaks at 474 and 524 cm^−1^ attributed to the ring δ_as_ (C–C) of Dabco moieties in CPF. XRD peaks of the prepared Ni-CPF and its microwave-transformed Ni-NCNT are shown in Figure 3b. In the case of Ni-CPF, two broad diffraction peaks at 10.2° and 14.4°, corresponding to Ni-CPF (CCDC No. 638866) [37] and two small humps at 33.8° and 60.2°, corresponding to the (108) and (200) planes of nickel (JCPDS No. 01–1260) can be observed. In the case of Ni-NCNT, the XRD spectra were dominated by a broad carbon peak at ~24.2° in addition to three sharp nickel related peaks at 13.5°, 37.5° and 63.3°. Since the EDS mapping images showed the extensive presence of nickel and nitrogen, XPS spectra were used to study the nature and extent of chemical moieties in Ni-NCNT. The freeware XPSPEAK ver. 4.1 was used to shape fit the Gaussian–Lorentzian type of high-resolution Ni 2p XPS spectra (Figure 3c), which shows four prominent peaks at 854.2, 961.23, 872.35 and 879.41 eV, corresponding to Ni 2p_3/2_ and its corresponding satellite peak and Ni 2p_1/2_ and its corresponding satellite peaks, respectively. 

The Ni 2p_3/2_ peak could be further deconvoluted into two peaks centered at 855.8 and 854.13 eV, corresponding to Ni^2+^ and Ni^3+^ moieties, respectively, which was further confirmed by the deconvoluted Ni 2p_½_ spectra, which shows two almost equal area doublets at 871.71 and 873.64 eV. Both satellite peaks can also be deconvoluted into two doublets, each at 860.55 and 862.35 in the Ni 2p_1/2_ region and 878.21 and 880.82 eV in the Ni 2p_3/2_ region, indicating that nickel moieties exist in a mixed-valence state of Ni^2+^ and Ni^3+^ [38]. High resolution deconvoluted N 1s XPS spectra were obtained to identify the electronic state of nitrogen in Ni-NCNT. The deconvoluted N 1s spectra in Figure 3d are dominated by three distinct peaks at 399.12, 399.98 and 401.1 eV, assigned to graphitic nitrogen as N-sp^3^ C [39], pyrrolic nitrogen [40] and pyridinic [41] nitrogen moieties, respectively.

**Figure 4:**
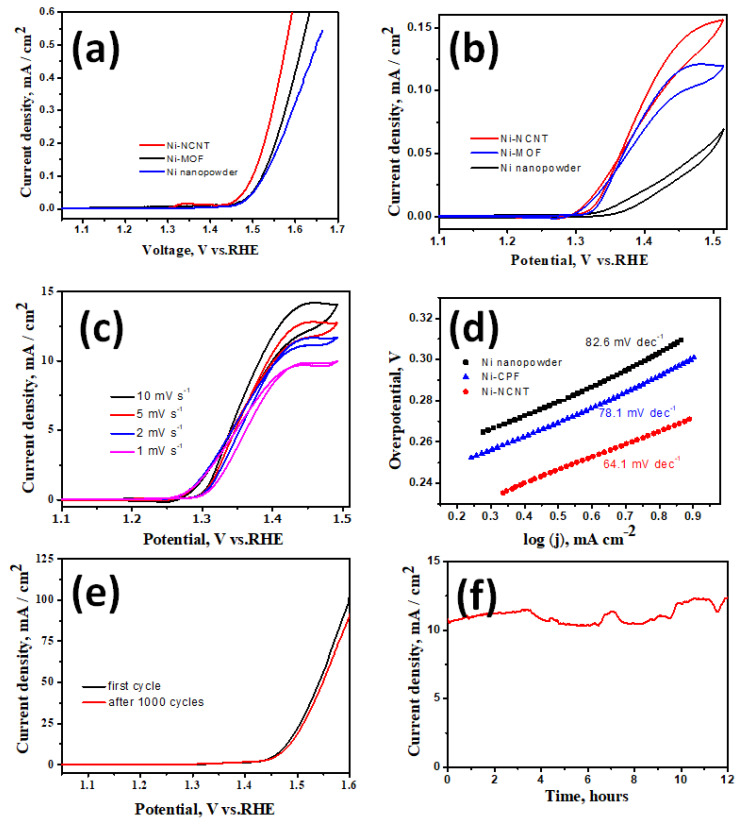
LSV curves of Ni-NCNT, Dabco Ni-CPF and NiO nanoparticles in 1 M KOH (**a**) and 1 M KOH with 0.33 M urea solution (**b**); LSV curves of Ni-NCNT at increasing scan rates (**c**); Tafel plot of Ni-NCNT, Dabco Ni-CPF and NiO in 1 M KOH with 0.33 M urea at a scan rate of 2 mV s^−1^ (**d**). LSV curves after 1000 cycles (**e**) and 12 h steady-state test (**f**).

Before evaluating the suitability of our newly developed Ni-NCNT as an electrocatalyst in the urea oxidation reaction, the electrodes were tested for water oxidation ability in a standard 1 M KOH solution. Among the three nickel-based catalysts investigated in this study, viz. NiO nanoparticles synthesized by the thermal treatment of nickel acetate (Representative SEM image in Figure S2a,b of supplementary file), Ni-CPF with Dabco as the organic ligand, and Ni-NCNT synthesized by microwave pyrolysis of Ni-CPF, Ni-NCNT showed the best performance in the water oxidation reaction when compared to NiO and Ni-CPF, which showed considerably inferior catalytic abilities (Figure 4a) and is comparable to reported nickel-based urea ORR catalysts (see Table S1 in supplementary file). This electrochemical behavior can be attributed to the different electronic structures of nickel in our newly synthesized Ni-NCNT, which shows unique synergistic effects with Ni nanoparticles embedded in defect-rich nitrogen-doped carbon nanotubes, which alters the electronic properties of nickel moieties, increases the conductivity and inhibits the agglomeration of Ni catalysts. The presence of nitrogen in the carbon nanotubes, as evident from EDS maps (Figure 1e) and XPS spectra (Figure 3d), acts effectively as electro-active sites and changes the electronic properties to synergistically improve the water oxidation activity [42,43]. Since confirming the utility of Ni-NCNT in water oxidation, we shifted our focus to study the electrocatalytic activity of NiO, Ni-CPF and Ni-NCNT in UOR in electrolytes consisting of 0.33 M urea in 1 M KOH. The value of 0.33 M urea was chosen based on previous studies, which showed that higher and lower concentrations of urea are vastly detrimental for catalysts [28]. CV curves of NiO, Ni-CPF and Ni-NCNT plotted in Figure 4b show that the highest urea oxidation current was obtained for the Ni-NCNT catalyst because of its high surface area active site exposure. It is well known that CPFs have high surface areas, but the electro-active nickel moieties are hybridized with organic ligands, which inhibit its electrocatalytic activity. However, in the case of Ni-NCNT, most of the nickel particles are anchored on the walls of the defect-rich carbon nanotubes, which makes them readily available for catalytic activity. The onset potential of Ni-NCNT for urea oxidation was also substantially lower than Ni-CPF and NiO, which shows that our developed Ni-NCNT can act as energy-efficient electrocatalysts for urea oxidation reactions. The kinetics for urea electro-oxidation of the Ni-NCNT electrocatalyst was further probed by studying the influence of scan rate on the electrocatalytic activity in 1 M KOH with 0.33 M urea solution (Figure 4c). Both the current density and ECSA increase for urea oxidation by increasing the scan rate, which indicates the dominance of a diffusion-controlled process.

Tafel slopes were used to evaluate the catalytic kinetics of the synthesized nickel-based catalysts. The polarization curves were recorded in 1 M KOH with 0.33 M urea at 2 mV s^−1^. As can be seen in Figure 4d, the Tafel slope of Ni-NCNT (64.1 mV dec^−1^) was substantially lower than those of Ni-CPF (78.1 mV dec^−1^) and NiO (82.6 mV dec^−1^). The smaller Tafel slope of the Ni-NCNT indicates an improved charge transfer rate and rapid kinetics during the urea oxidation process due to the synergy between Ni-N-C. The urea electro-oxidation process is a very complex phenomenon in which a six-electron process occurs, and some controversy remains regarding the exact mechanism. Of the many proposed mechanisms, the most widely accepted is that electrochemical oxidation of Ni^3+^ and Ni^2+^ to nickel oxyhydroxides generates the products CO_2_, N_2_, and H_2_O. The stability of the Ni-NCNT electrocatalyst was also confirmed by comparing the polarization curve before and after consecutive scanning for 1000 cycles (Figure 4e) and the steady-state test for 12 h (Figure 4f). After 1000 cycles, there was a marginal decrease of 12% in the current density, whereas the steady-state tests showed a slight 3% increase in current density after 12 h of continuous operation. The high stability can be attributed to the protective carbon coating onto the nickel nanoparticles, as evident from the high-resolution TEM image exhibited in Figure 1d, which shows that the nickel moieties on the walls of NCNT are core-shell structured with a few layers of graphene encapsulating the irregular-shaped nickel nanoparticles. This protective graphene coating acts as a good diffusion barrier, inhibiting the degradation of nickel in the harsh alkaline electrolyte. 

## 4. Conclusions

In summary, nickel-nitrogen doped carbon nanotubes prepared by fast and facile microwave pyrolysis of Dabco-based Ni-CPF were studied as electrocatalysts for urea electro-oxidation. Morphological analysis of Ni-NCNT, as studied by SEM and TEM techniques, showed core-shell nickel nanoparticles anchored on micrometer-long, bamboo-shaped and defect-rich nanotubes. The EDS map showed that the majority of nitrogen moieties were embedded in the carbon nanotubes with only a minor portion attached to nickel. The outstanding electrocatalytic activity, stability and tolerance towards urea oxidation indicated promising applications of CPF-derived Ni-NCNT in electrochemical urea removal and fuel cell technology.

## Data Availability

The data presented in this study are available on request from the corresponding author.

## Supplementary Materials

The following supporting information can be downloaded at: https://www.mdpi.com/article/10.3390/ma15062048/s1, Figure S1: Representative SEM images of Dabco based Ni MOFs; Figure S2: Representative SEM images of Ni nanoparticles synthesized by thermal degradation of nickel acetate; Table S1: Comparison of the urea electrochemical activity for some nickel based catalysts [44,45,46,47,48,49].
