# Supplementary material for: Coordination Polymer Framework-Derived Ni-N-Doped Carbon Nanotubes for Electro-Oxidation of Urea
Source: Materials (Basel). 2022 Mar 10;15(6):2048. doi: 10.3390/ma15062048 (PMC8955885; doi:10.3390/ma15062048)
Supplement: Supplementary file 1 [file materials-15-02048-s001.zip › materials-1585655-supplementary.pdf]

materials

MDPI

Supplementary Material

Coordination Polymer Framework-Derived Ni-N-Doped Carbon Nanotubes for Electro-Oxidation of Urea

Vadahanambi Sridhar [1] and Hyun Park [1,2,*]

[1] Global Core Research Centre for Ships and Offshore Plants (GCRC-SOP), Pusan National University, Busan 46241, Korea; sridhar@pusan.ac.kr
[2] Department of Naval Architecture and Ocean Engineering, Pusan National University, Busan 46241, Korea
* Correspondence: hyunpark@pusan.ac.kr; Tel.: +82-51-510-2730

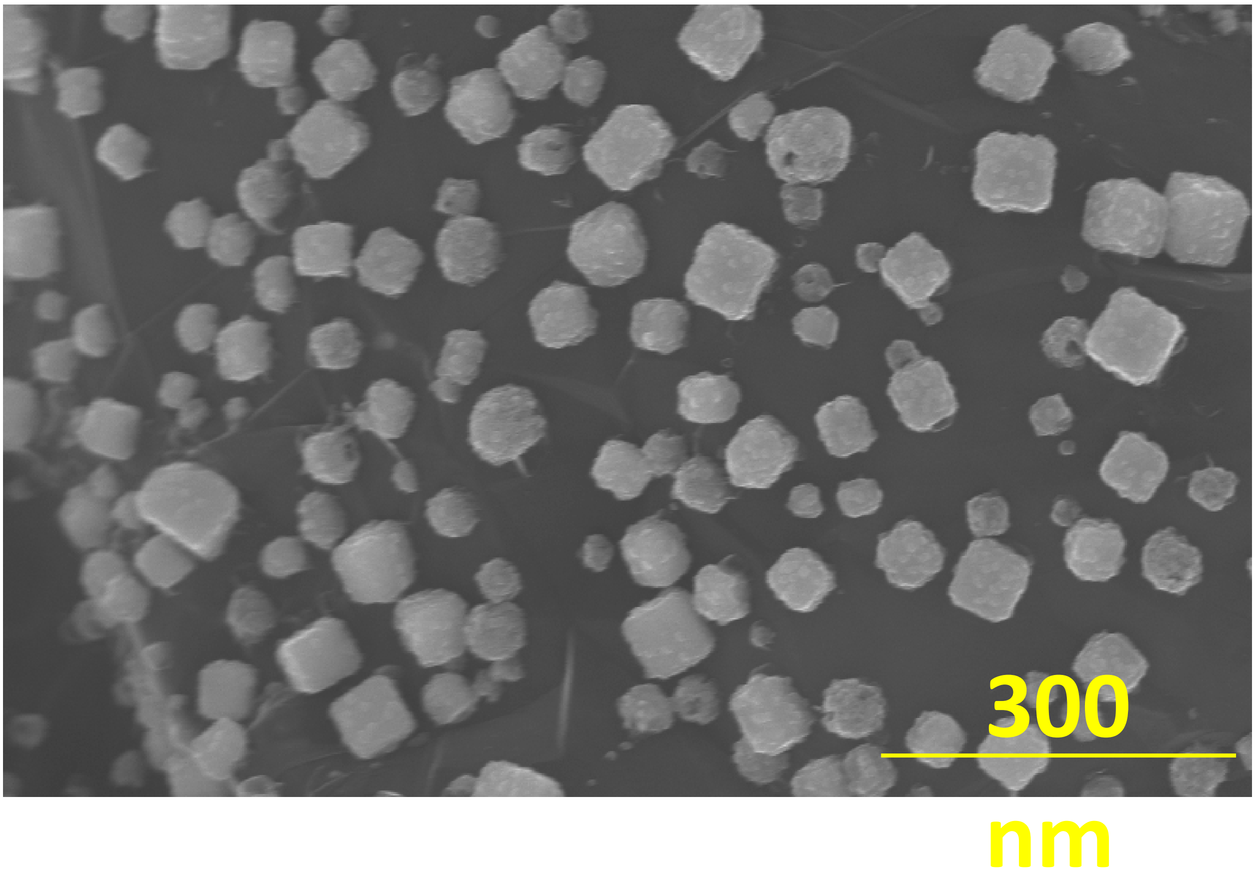

Figure S1. Representative SEM images of Dabco based Ni MOFs.

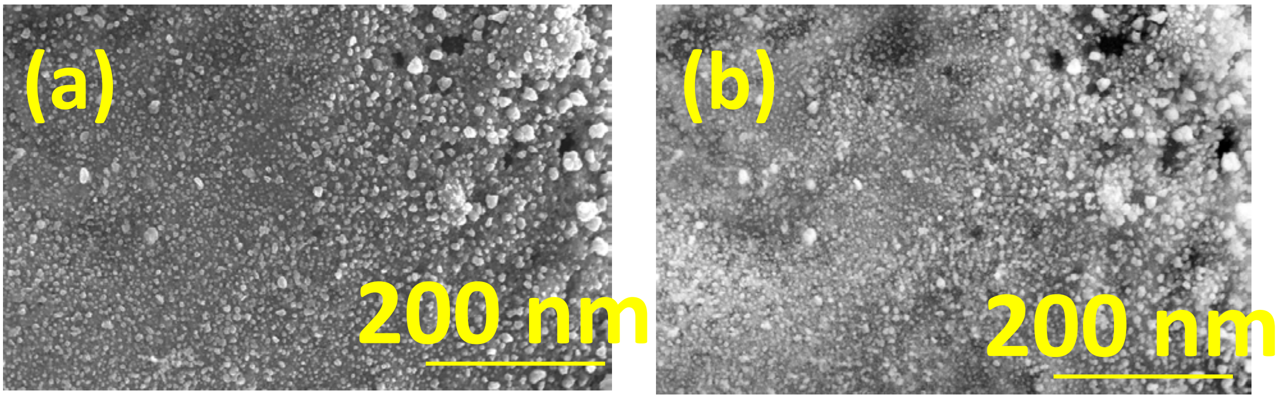

Figure S2. Representative in-lens (**a**) and secondary electron emission (**b**) SEM images of Ni nanoparticles synthesized by thermal degradation of nickel acetate.

Table S1. Comparison of the urea electrochemical activity for some nickel based catalysts.

S. No	Material	Morphology	Potential, V vs. RHE	Reference
1	Ni_3N NA/CC	Nickel nitride nanosheets on carbon cloth	1.34	[1]
2	Ni_2P/NF	Nickel phosphide flakes on carbon cloth	1.37	[2]
3	$Ni(OH)_2$ nanotube-NF	No morphological study reported	1.41	[3]
4	NiO nanosheet array	Nickel oxide on nickel foam	1.38	[4]
5	ERGO-Ni	Nickel oxide nanoparticles on reduced graphene oxide	1.45	[5]
6	$Ni(OH)_2$ nanocube	Porous Nickel hydroxide	1.55	[6]
7	Ni-NCNT	Nickel nanoparticles embedded in nitrogen doped carbon nanotubes	1.35	This work

References

1. Liu: Q.; Xie, L.; Qu, F.; Liu, Z.; Du, G.; Asiri, A.M.; Sun, X. A porous Ni3N nanosheet array as a high-performance non-noble-metal catalyst for urea-assisted electrochemical hydrogen production. *Inorg. Chem. Front.* **2017**, *4*, 1120–1124, doi:10.1039/c7qi00185a.
2. Liu, D.; Liu, T.; Zhang, L.; Qu, F.; Du, G.; Asiri, A.M.; Sun, X. High-Performance urea electrolysis towards less energy-intensive electrochem-ical hydrogen production using a bifunctional catalyst electrode. *J Mater Chem A* **2017**, *5*, 3208–3213.
3. Ji, R.Y.; Chan, D.S.; Jow, J.J.; Wu, M.S. Formation of open-ended nickel hydroxide nanotubes on three-dimensional nickel framework for enhanced urea electrolysis. *Electrochem. Commun.* **2013**, *29*, 21–24, doi:10.1016/j.elecom.2013.01.006.
4. Wu, M.S.; Lin, G.W.; Yang, R.S. Hydrothermal growth of vertically-aligned ordered mesoporous nickel oxide nanosheets on three-dimensional nickel framework for electrocatalytic oxidation of urea in alkaline medium. *J. Power Sources* **2014**, *272*, 711–718, doi:10.1016/j.jpowsour.2014.09.009.
5. Wang, D.; Yan, W.; Vijapur, S.H.; Botte, G.G. Electrochemically reduced graphene oxide–nickel nanocomposites for urea electrolysis. *Electrochimica Acta* **2013**, *89*, 732–736, doi:10.1016/j.electacta.2012.11.046.
6. Wu, M.S.; Ji, R.Y.; Zheng, Y.R. Nickel hydroxide wlectrode with a monolayer of nanocup arrays as an effective electrocatalyst for enhanced electrolysis of urea. *Electrochim Acta* **2014**, *144*, 194–199.
